# *Ancylostoma ceylanicum* Hookworm in Myanmar Refugees, Thailand, 2012–2015

**DOI:** 10.3201/eid2408.180280

**Published:** 2018-08

**Authors:** Elise M. O’Connell, Tarissa Mitchell, Marina Papaiakovou, Nils Pilotte, Deborah Lee, Michelle Weinberg, Potsawin Sakulrak, Dilok Tongsukh, Georgiette Oduro-Boateng, Sarah Harrison, Steven A. Williams, William M. Stauffer, Thomas B. Nutman

**Affiliations:** National Institutes of Health, Bethesda, Maryland, USA (E.M. O’Connell, G. Oduro-Boateng, S. Harrison, T.B. Nutman);; Centers for Disease Control and Prevention, Atlanta, Georgia, USA (T. Mitchell, D. Lee, M. Weinberg, W.M. Stauffer);; Smith College, Northampton, Massachusetts, USA (M. Papaiakovou, N. Pilotte, S.A. Williams);; University of Massachusetts, Amherst, Masschusetts, USA (N. Pilotte, S.A. Williams);; International Organization for Migration, Mae Sot, Thailand (P. Sakulrak, D. Tongsukh);; University of Minnesota Medical School, Minneapolis, Minnesota, USA (W.M. Stauffer)

**Keywords:** hookworm, *Ancylostoma*, *Ancylostoma ceylanicum*, *Ancylostoma duodenale*, *Necator americanus*, soil-transmitted helminths, refugees, Myanmar, Thailand, Burmese, United States, parasites, albendazole, β-tubulin SNP, SNP200, SNP167, neglected tropical disease, benzimidazole, zoonoses, eosinophils, hemoglobin, cure rate

## Abstract

During 2012–2015, US-bound refugees living in Myanmar–Thailand border camps (n = 1,839) were surveyed for hookworm infection and treatment response by using quantitative PCR. Samples were collected at 3 time points: after each of 2 treatments with albendazole and after resettlement in the United States. Baseline prevalence of *Necator americanus* hookworm was 25.4%, *Ancylostoma duodenale* 0%, and *Ancylostoma ceylanicum* (a zoonosis) 5.4%. Compared with *N. americanus* prevalence, *A. ceylanicum* hookworm prevalence peaked in younger age groups, and blood eosinophil concentrations during *A. ceylanicum* infection were higher than those for *N. americanus* infection. Female sex was associated with a lower risk for either hookworm infection. Cure rates after 1 dose of albendazole were greater for *A. ceylanicum* (93.3%) than *N. americanus* (65.9%) hookworm (p<0.001). Lower *N. americanus* hookworm cure rates were unrelated to β-tubulin single-nucleotide polymorphisms at codons 200 or 167. *A. ceylanicum* hookworm infection might be more common in humans than previously recognized.

## Abstract 2

This hookworm, uncommonly found in humans, has a higher cure rate than that for *Necator americanus* hookworm.

Hookworm infection affects >470 million persons worldwide (*1*). Childhood infection has been associated with growth stunting, severe anemia, and iron deficiency (*2*,*3*). *Ancylostoma duodenale* and *Necator americanus* hookworms are believed to be the most prevalent species that infect humans. Infections with these species are acquired by transdermal penetration of the hookworm larvae or by the fecal-oral route (*A. duodenale* hookworm only), and infection is limited to humans (*4*). Dogs and cats infected with *Ancylostoma ceylanicum* hookworm have been found in close association with human populations (*5*–*9*). With use of molecular techniques, an increased number of human *A. ceylanicum* hookworm infections have been documented in parts of Asia and the Solomon Islands (*10*–*13*).

Hookworms and other soil-transmitted helminths are neglected tropical pathogens targeted for worldwide control by the World Health Organization by 2030 (*14*). This goal is being pursued through the mass administration of benzimidazole compounds (i.e., albendazole, mebendazole). However, the ubiquitous use of a single class of drug in both human and veterinary medicine has raised concern for the emergence of drug resistance (*15*–*20*). We report hookworm infection and cure rates in a large cohort of US-bound refugees from Myanmar residing in camps in Thailand along the Myanmar–Thailand border and assess the presence of β-tubulin mutations that could confer drug resistance among persons with persistent infection.

## Materials and Methods

### Recruitment of Participants and Sample Collection

**Figure 1:**
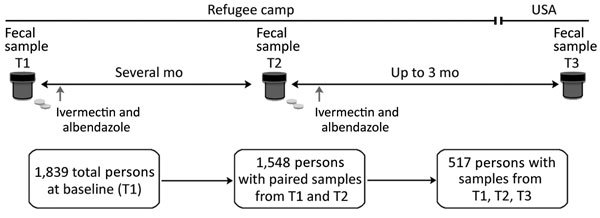
Study design showing collection of fecal samples from and treatment of US-bound Myanmar refugees for hookworm infection, Thailand, 2012–2015. Myanmar refugees (n = 2,004) from 3 camps in Thailand (Mae La [camp 1], Mae Ra Ma Luang [camp 2], Mae La Oon [camp 3]) along the Myanmar–Thailand border were recruited to donate fecal samples and receive treatment (ivermectin and albendazole) for parasitic infections. T1 was the time of the resettlement medical exam, T2 occurred before camp departure, and T3 was a time after resettlement in the United States. Albendazole and ivermectin were given immediately after fecal collection at T1 and T2. T1, time point 1; T2, time point 2; T3, time point 3.

This investigation was part of a larger program involving refugees living in camps along the Myanmar–Thailand border that was conducted by the Centers for Disease Control and Prevention during 2012–2015 (*21*). In our analysis, we included 1,839 (92%) of the 2,004 refugees >6 months of age from this cohort who provided fecal samples. (Figure 1; Technical Appendix). Fecal samples were collected at time point 1 (T1, during the required medical examination for US resettlement), T2 (before departing the refugee camp), and T3 (after US resettlement). All refugees were offered albendazole after fecal specimens were collected to treat presumptive infection with helminths.

### Molecular Detection of Hookworm Species

We performed stool extraction (Technical Appendix) as previously described (*22*). For the initial 233 samples, we performed quantitative PCR (qPCR) with primer-probe sets Ad1 (for amplifying *A. duodenale*) and Na (for amplifying *N. americanus*) (Table 1) (*23*,*24*). Then, we switched to a more sensitive primer-probe set targeting a repetitive element in *A. duodenale* (Ad2) (*24*). When qPCR with the Ad2 primer-probe set failed to amplify the samples positive by qPCR with Ad1, we subjected 7 discordant samples to PCR restriction fragment length polymorphism, as previously described (*26*). We also sequenced these PCR products using standard (Sanger) sequencing technology.

**Table 1:** Primer-probe sets used to determine genotypes of hookworms present in US-bound Myanmar refugees in camps along Myanmar–Thailand border, Thailand, 2012–2015*

Genome target/primer-probe set name, sequence type	Sequence	Final concentration, nmol/L	Reference
ITS2/Ad1			(*23*)
Forward primer	5′-GAATGACAGCAAACTCGTTGTTG-3′	900	
Reverse primer	5′-ATACTAGCCACTGCCGAAACGT-3′	900	
Probe	5′-ATCGTTTACCGACTTTAG-3′	250	
Repetitive element/Ad2			(*24*)
Forward primer	5′-GTATTTCACTCATATGATCGAGTGTTC-3′	900	
Reverse primer	5′-GTTTGAATTTGAGGTATTTCGACCA-3′	900	
Probe	5′-TGACAGTGTGTCATACTGTGGAAA-3′	250	
Repetitive element/Ac			(*25*)
Forward primer	5′-CAAATATTACTGTGCGCATTTAGC-3′	900	
Reverse primer	5′-GCGAATATTTAGTGGGTTTACTGG-3′	900	
Probe	5′-CGGTGAAAGCTTTGCGTTATTGCGA-3′	250	
Repetitive element/Na			(*24*)
Forward primer	5′-CCAGAATCGCCACAAATTGTAT-3′	900	
Reverse primer	5′-GGGTTTGAGGCTTATCATAAAGAA-3′	900	
Probe	5′-CCCGATTTGAGCTGAATTGTCAAA-3′	250	
SNP200			This paper
Forward primer	5′-AATGCTACACTCTCTGTTCACCAGTT-3′	900	
Reverse primer	5′-CGGAAGCAGATATCATACAAAGCTT-3′	900	
Wild-type FAM probe/mutant VIC probe†	5′-AATACAGATGAGACCT(T/A)CT-3′	166; 231	
SNP167			This paper
Forward primer	5′-TCGGGAAGAATACCCTGATAGAAT-3′	900	
Reverse primer	5′-CTTTTGCTCTTATTTCCATCAATAGGA-3′	900	
Wild-type FAM probe/mutant VIC probe†	5′-TGTCCTCGT(T/A)TTCC-3′	125; 350	

*Ac, *Ancylostoma ceylanicum* set; Ad1, *A. duodenale* set 1; Ad2, *A. duodenale* set 2; ITS2, internal transcribed spacer 2; Na, *Necator americanus* set; SNP, single-nucleotide polymorphism. †FAM and VIC probes (Integrated DNA Technologies, Skokie, IL, USA).

### Definitions

Participants whose fecal samples were positive for hookworm DNA by qPCR and became negative at the immediate next time point were considered cured. Those whose fecal samples were negative for hookworm DNA by qPCR but then positive the immediate next time point were considered to have a newly acquired infection. We defined persistent infection as having detectable hookworm DNA at 2 successive time points.

### Single-Nucleotide Polymorphism Detection in *N. americanus*–Positive Samples

For the participants who were positive for *N. americanus* hookworm at all 3 time points, we tested fecal samples from T1 and T3 for β-tubulin single-nucleotide polymorphisms (SNPs) at codon 200. We also tested refugee T3 fecal samples for SNP167 by using an allele-specific real-time PCR approach with common primers and SNP-specific probes (Table 1) and defined heterozygosity and homozygosity of SNPs on the basis of change in the cycle threshold, similar to previously described methods (*27*–*29*) (Technical Appendix).

### Statistical Analyses

Unless stated otherwise, we used the geometric mean to measure central tendency. We determined the odds ratios (ORs) of risk factors for infection with *A. ceylanicum* and *N. americanus* hookworms by using a generalized linear model that used overdispersion with binomial distribution and logit link. We performed a maximum likelihood analysis using JMP 12.0.1 (https://www.jmp.com/en_us/home.html). We used these models to test the following parameters: sex, camp, age (infants and toddlers <2 years of age, children 2–18 years of age, and adults >18 years of age), and co-infections (Technical Appendix). The cutoff of <2 years of age for infants and toddlers was chosen because, after this age, the rates of mouthing, a prominent cause of fecal-oral contamination, decrease (*30*,*31*). We performed cure rate comparisons using a 2-tailed Fisher exact test. We compared the geometric mean eosinophil and hemoglobin concentrations of those monoinfected with *A. ceylanicum* or *N. americanus* hookworm (excluding co-infections involving both hookworms and *Strongyloides stercoralis*, *Ascaris lumbricoides*, and *Trichuris trichiura* roundworms) by the Mann-Whitney test using Prism GraphPad 6.0e (https://www.graphpad.com/scientific-software/prism/).

## Results

A total of 4,330 fecal samples from 1,839 refugees underwent DNA extraction and multiparallel qPCR. After excluding 4 participants positive for hookworm at T1 who did not receive albendazole, the number of participants with T1-T2 paired samples totaled 1,548; we had samples from all 3 time points for 517 participants. The geometric mean time between T1 and T2 sample collections was 188 (range 48–1,013) days and between T2 and T3 was 46.2 (range 14–413) days. Baseline hookworm infection (any type) in this population was high, ranging from 25.9%–32.8% depending on the camp of residence (Table 2).

**Table 2:** Baseline characteristics of 1,839 US-bound Myanmar refugees in camps along Myanmar–Thailand border, by camp, Thailand, 2012–2015

Characteristic	Mae La, camp 1	Mae La Oon, camp 2	Mae Ra Ma Luang, camp 3
Total participants, no.	549	667	623
Sex, no. (%)			
F	261 (47.5)	327 (49.0)	304 (48.8)
M	288 (52.5)	340 (51.0)	319 (51.2)
Age, y, mean (range)	20.8 (0.6–79.9)	19.3 (0.86–88.9)	18.9 (0.55–83.1)
Any hookworm infection, no. (%)	180 (32.8)	173 (25.9)	182 (29.2)
Hemoglobin, g/L, geometric mean (range)	130 (71–179)	127 (71–180)	126 (71–183)
Eosinophil concentration, × 10^8^ cells/L, geometric mean (range)*	3.56 (0.01–55.3)	4.27 (0.01–107)	5.21 (0.01–178)

*Eosinophil concentration reference range: 0–4.5 × 10^8^ cells/L.

### Detection of *A. ceylanicum*

**Figure 2:**
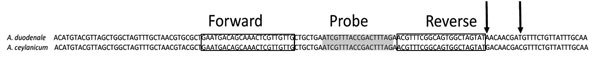
Partial internal transcribed spacer 2 (ITS2) sequences of *Ancylostoma duodenale* and *A. ceylanicum* hookworms. Boxes indicate the location of forward and reverse primer binding; gray shading indicates the location of probe binding. These regions of the *A. duodenale* and *A. ceylanicum* ITS2 are identical. The locations where the ITS2 sequences differ (arrows) fall outside of the primer and probe binding regions.

When a highly sensitive primer-probe set specific to the *A. duodenale* genome (Ad2) was used on samples positive for *Ancylostoma* DNA by qPCR with primer-probe set Ad1, none was positive. Because of concern that Ad1 might enable the cross-amplification of other *Ancylostoma* spp., we performed seminested PCR and *Mva*I and *Psp*1406I digestion with 7 samples positive by Ad1 but negative by Ad2. Results from restriction fragment length polymorphism PCR indicated the presence of *A. ceylanicum* hookworm (Technical Appendix Figure); these results were further confirmed by sequencing (98% identity to *A. ceylanicum* ribosomal sequence [GenBank accession no. LC036567]). The internal transcribed spacer 2 regions of the *A. duodenale* and *A. ceylanicum* genomes, which the Ad1 primer-probe set aligned with, are identical (Figure 2). Cross-species identification with this primer-probe set has been previously predicted (*23*), although not previously demonstrated in the literature.

We then determined the prevalence of *A. ceylanicum* hookworm among the refugee population using a primer-probe set specific to a repetitive DNA element in the *A. ceylanicum* genome (Ac; Table 1). When using Ac, the total number of *A. ceylanicum*–positive samples increased from 106 (using the Ad1 set) to 124 (using the Ac set). We tested or retested these samples (n = 124) by qPCR using the Ad2 primer-probe set, and 0% were positive. All samples positive by qPCR with Ad1 were positive by qPCR with Ac.

### Response to Treatment

**Figure 3:**
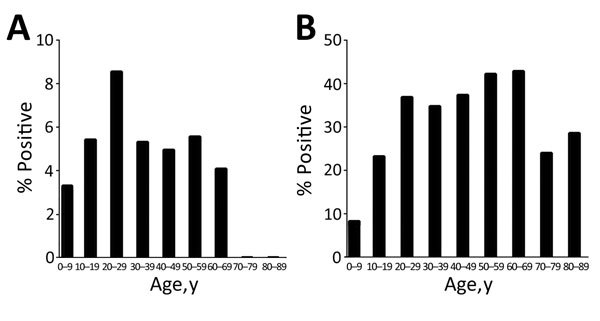
Baseline prevalence of hookworm infections in 1,839 US-bound Myanmar refugees at 3 camps along the Myanmar–Thailand border, by age group, Thailand, 2012–2015. A) *Ancylostoma* *ceylanicum* hookworm. B) *Necator americanus* hookworm.

Baseline prevalence of *N. americanus* (26.3%) hookworm was higher than that of *A. ceylanicum* (5.3%) hookworm among all participants (n = 1,839); likewise, prevalence of *N. americanus* (25.4%) species was higher than that of *A. ceylanicum* (5.4%) species among all participants who gave paired T1-T2 fecal samples (n = 1,548) (Table 3). Refugees in their sixth (50–59 years) and seventh (60–69 years) decades of life had the highest *N. americanus* hookworm prevalence (>40% positive), and refugees in their third decade of life had the highest *A. ceylanicum* hookworm prevalence (9%) (Figure 3).

**Table 3:** Hookworm prevalence and cure rate in US-bound Myanmar refugees in camps along Myanmar–Thailand border with paired samples, by time point, Thailand, 2012–2015*

Species	T1, n = 1,548		T2, n = 1,548			T3, n = 517	
	Baseline prevalence, no. (%)		Cure, no. (%)	Persistent infection, no. (%)		Cure, no. (%)	Persistent infection, no. (%)
*Ancylostoma ceylanicum*	83/1,548 (5.4)		77/83 (92.8)	6/83 (7.2)		7/7 (100)	0
*Necator americanus*	393/1,548 (25.4)		271/393 (69)	122/393 (31)		21/50 (42)	29/50 (58)

*The geometric mean time from T1 to T2 was 188.2 (range 48–1,013) d and from T2 to T3 was 46.2 (range 14–413) d. T1, time point 1; T2, time point 2; T3, time point 3.

*A. ceylanicum* infection had a higher cure rate than did *N. americanus* infection (Table 3); 92.8% of *A. ceylanicum* hookworm–infected refugees were cured by T2, despite the relatively long time that elapsed between T1 and T2. Of the samples paired for T2 and T3, all 7 *A. ceylanicum* hookworm–infected refugees at T2 were cured by T3. At T2, the *N. americanus* infection cure rate was 69%, and after the second administration of albendazole at T3, 42% (21/50) were cured, resulting in 29 participants with persistent *N. americanus* infection at resettlement in the United States. Combining cure rates across all time points, the overall *A. ceylanicum* hookworm cure rate was 93.3% (84/90), higher than that for *N. americanus* hookworm (65.9%, 292/443; p<0.001).

**Figure 4:**
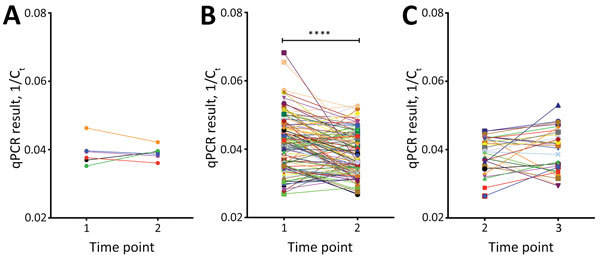
Change in relative quantities of *Ancylostoma ceylanicum* and *Necator americanus* hookworm genomic DNA in fecal samples from US-bound Myanmar refugees at 3 camps along the Myanmar–Thailand border after treatments with albendazole and ivermectin, Thailand, 2012–2015. Fecal samples were collected at 3 time points: time point 1 (T1, baseline), time point 2 (T2, after first treatment), and time point 3 (T3, after second treatment). Quantities were expressed as 1/C_t_ and differences were assessed by paired *t*-test. A) *A. ceylanicum* genomic DNA relative quantities in those who were persistently infected after first treatment (n = 6). The difference between T1 and T2 was not significant. B) *N. americanus* genomic DNA relative quantities in those who were persistently infected after first treatment (n = 122). The relative genomic DNA quantity was significantly reduced in those who remained infected with *N. americanus* hookworm after first treatment. C) *N. americanus* genomic DNA relative quantities in those who were persistently infected after second treatment (n = 29). No significant change in *N. americanus* relative quantity was found in those persistently infected after the second treatment. The geometric mean time from T1 to T2 was 188.2 (range 48–1,013) d and from T2 to T3 was 46.2 (range 14–413) d. ****p<0.0001. C_t_, cycle threshold; qPCR, quantitative PCR.

In total, 151 refugees had persistent *N. americanus* infections, and 6 refugees had persistent *A. ceylanicum* infections. At T2, the hookworm genomic DNA relative quantity in fecal samples of those with persistent *N. americanus* infections (n = 122) decreased significantly (p<0.001) (Figure 4). Of the 6 persons with persistent *A. ceylanicum* infections at T2, 4 (67%) had a decrease and 2 (33%) an increase in hookworm genomic DNA in their fecal samples. Those infected with *N. americanus* hookworm at T2 and T3 (n = 29) had no significant decrease in parasite genomic DNA relative quantity at T3, and 26 refugees were positive for *N. americanus* hookworm at all 3 time points.

We detected a small number of newly acquired hookworm infections. Of the 1,548 participants with T1-T2 paired samples, we detected 15 (≈1%) new *A. ceylanicum* infections and 36 (2.3%) new *N. americanus* infections (Table 4). Of the 517 participants with T2-T3 paired samples, we detected 3 (0.58%) new *A. ceylanicum* infections and 13 (2.5%) new *N. americanus* infections.

**Table 4:** New hookworm infections acquired by US-bound Myanmar refugees in camps along Myanmar–Thailand border, by time point, Thailand, 2012–2015*

Species	T2, n = 1,548, no. (%)	T3, n = 517, no. (%)
*Ancylostoma ceylanicum*	15 (0.97)	3 (0.58)
*Necator americanus*	36 (2.3)	13 (2.5)

*T2, time point 2; T3, time point 3.

### Risk Factors Associated with *N. americanus* and *A. ceylanicum* Infection

A generalized linear model was used to assess whether coincident helminth or protozoa infection, age, sex, or camp affected the risk for infection with *N. americanus* or *A. ceylanicum* hookworm at T1 (Table 5). The *y*-intercept for the model of *N. americanus* hookworm was 2.4 (95% CI 2.14–2.69) and for *A. ceylanicum* hookworm 3.24 (95% CI 2.77–3.74). The strongest predictors (ORs >1.5 and p values <0.0001) of *N. americanus* infection were adult age (OR 3.83), *T. trichiura* infection (OR 1.90), and *A. lumbricoides* infection (OR 1.71). Female participants had a reduced odds of *N. americanus* infection (OR 0.68; p<0.0001). *N. americanus* infection was the only 1 of the 5 infections assessed that was associated with *A. ceylanicum* co-infection (OR 2.08; p = 0.0018); female sex (OR 0.57; p<0.0001) and residence in camp 1 (Mae La, OR 0.69; p = 0.03) were associated with reduced odds of *A. ceylanicum* infection.

**Table 5:** Characteristics of US-bound Myanmar refugees in camps along Myanmar–Thailand border associated with increased risk for infection with *Ancylostoma ceylanicum* or *Necator americanus* hookworm, Thailand, 2012–2015*

Category	*N. americanus*			*A. ceylanicum*	
	Odds ratio	p value		Odds ratio	p value
Children compared with infants and toddlers	8.17	0.0025		NS	NS
Adults compared with children	3.83	<0.0001		NS	NS
*Ancylostoma ceylanicum* infection	2.08	0.0017		NA	NA
*Trichuris trichiura* infection	1.90	<0.0001		NS	NS
*Entamoeba histolytica* infection	1.79	0.0173		NS	NS
*Ascaris lumbricoides* infection	1.71	<0.0001		NS	NS
Residence at camp 1, Mae La	1.27	0.0039		0.69	0.0303
Female sex	0.68	<0.0001		0.57	<0.0001
*Necator americanus* infection	NA	NA		2.08	0.0018

*We built the following factors into the model: other infections (*Strongyloides* stercoralis, *A. lumbricoides*, *E. histolytica*, *Cryptosporidium* spp., *T. trichiura*, *Giardia* duodenalis); camp (1, 2, 3); age (infants and toddlers <2 years of age, children 2–18 years of age, adults >18 years of age); and sex. Associations with *Cryptosporidium* spp. and *G.* *duodenalis* were not significant. We used a generalized linear model that used overdispersion with binomial distribution and logit link, and we performed maximum-likelihood analysis using JMP 12.0.1 (https://www.jmp.com/en_us/home.html). NA, not applicable; NS, not significant.

We used a similar model that took age, sex, and camp into account to compare participants who cleared their infection with *N. americanus* hookworm after 1 treatment (n = 290) with those who did not clear infection after 2 treatments (n = 26). Female sex, but not age or camp, was associated with clearance after a single treatment (OR 2.14, 95% CI 1.23–4.44; p = 0.0045).

### *N. americanus* Benzimidazole Resistance and β-Tubulin SNP Changes

Because 26 participants were positive for *N. americanus* hookworm at all 3 time points despite 2 courses of albendazole treatment, benzimidazole drug resistance was a concern. In total, 19 of 26 persistently infected participants had T1 fecal samples available for DNA reextraction and SNP200 testing; 3 samples showed no amplification, and 16 were wild type. We then performed DNA reextraction and SNP200 and SNP167 testing with the T3 fecal samples available (n = 24). Only 11 of 24 samples had sufficient quantities of *N. americanus* DNA (>150 pg/µL) to be amplified. All samples were positive for wild-type SNP200 and SNP167. None were homozygous or heterozygous for mutant alleles. Thus, no alterations in the β-tubulin gene could be detected at codons 167 or 200 to account for drug resistance.

An additional 213 samples (from multiple time points) had sufficient *N. americanus* DNA to test SNP200 variation further; 173 of 213 samples were evaluable by allelic discrimination qPCR. All were homozygous wild type for SNP200.

### Blood Cell Concentration Differences Between *N. americanus* and *A. ceylanicum* Infections

**Figure 5:**
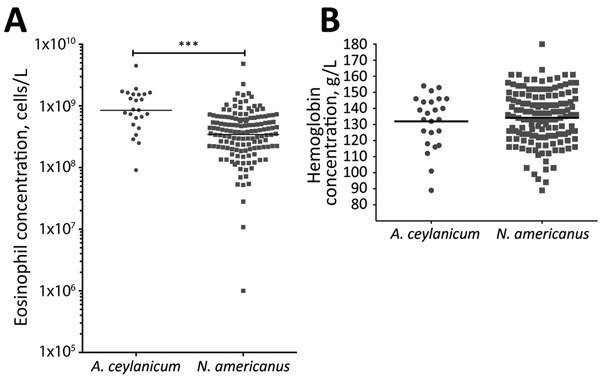
Eosinophil and hemoglobin concentrations in US-bound Myanmar refugees at 3 camps along the Myanmar–Thailand border who were monoinfected with *Ancylostoma ceylanicum* (n = 24) or *Necator americanus* (n = 143) hookworm at baseline, Thailand, 2012–2015. Those co-infected with both hookworms or *Strongyloides stercoralis*, *Ascaris lumbricoides*, or *Trichuris trichiura* roundworms were excluded from analysis. Horizontal line indicates geometric mean. Significance was calculated by Mann-Whitney test. A) The geometric mean eosinophil cell concentration was significantly higher in those with *A. ceylanicum* monoinfection (8.49 × 10^8^ cells/L, 95% CI 5.98–12.0 × 10^8^ cells/L) than those with *N. americanus* monoinfection (3.44 × 10^8^ cells/L, 95% CI 2.92–4.05 × 10^8^ cells/L) (***p<0.001). B) The geometric mean hemoglobin level of the 2 groups was not significantly different.

We further evaluated refugees with either *N. americanus* (n = 143) or *A. ceylanicum* (n = 24) hookworm monoinfections; participants co-infected with both hookworms or other soil-transmitted helminths (i.e., *S. stercoralis*, *A. lumbricoides*, and *T. trichiura* roundworms) were excluded. Peripheral blood eosinophil concentrations were significantly higher (p<0.001) in those with *A. ceylanicum* monoinfections (geometric mean 8.49 × 10^8^ cells/L, 95% CI 5.98–12.04 × 10^8^ cells/L) than those with *N. americanus* monoinfections (geometric mean 3.44 × 10^8^ cells/L, 95% CI 2.92–4.05 × 10^8^ cells/L) (Figure 5, panel A). The hemoglobin levels did not differ between those with only *A. ceylanicum* and those with only *N. americanus* infections (Figure 5, panel B).

## Discussion

*A. ceylanicum* hookworm is increasingly being recognized as a pathogen in humans, particularly in Southeast Asia (*32*). *A. ceylanicum* is the only hookworm species known to achieve patency in both humans and other animals (e.g., dogs and cats) (*33*). In this group of US-bound refugees from Myanmar, *N. americanus* infection was the most prevalent hookworm infection at all 3 time points tested (25.4% at baseline); *A. ceylanicum* was the only other hookworm species found, with a baseline prevalence of 5.4%. Because *A. ceylanicum* infection has been described in both Thailand (*7*,*10*) and Myanmar (*11*,*34*), whether these persons were infected in their home country or in the camps in Thailand is unknown. Camp allocation seemed to have an effect on infection status for both hookworms; camps 2 and 3 imparted a higher risk for *A. ceylanicum* hookworm acquisition, and camp 1 had a higher rate of *N. americanus* infection. Evidence of newly acquired infections for both hookworms at T2 and T3 (Table 4) indicates that active transmission of both species was ongoing at these 3 camps. However, whether the majority of hookworm infections at baseline were acquired prior to entry or while residing in the camps is unknown. Differential infection rates at the 3 camps might reflect environmental and hygiene conditions in the camps, the historical exposure of the persons at these camps, or other factors. Of note, a risk factor for infection with either *A. ceylanicum* or *N. americanus* hookworm was infection with the other hookworm (Table 5). This finding contrasts with previous surveys showing that *A. ceylanicum* and *N. americanus* co-infections are rare (*26*,*32*). Although *A. ceylanicum* infection by the fecal-oral route is thought to be possible (*33*), walking barefoot has been shown to be a major risk factor for *A. ceylanicum* and *N. americanus* hookworm infections (*35*). Thus, the high propensity for co-infection in the population we evaluated suggests a similar mode of transmission, namely transdermal penetration, for both hookworms.

Despite possible co-transmission, those infected with *A. ceylanicum* hookworm had slightly different risk factors than those infected with *N. americanus* hookworm. *A. ceylanicum* hookworm prevalence peaked in the third decade of life, compared with *N. americanus* prevalence, which peaked in the sixth and seventh decades. However, *N. americanus* prevalence remained high for many decades of life (20–69 years of age).

*T. trichiura* roundworm, *Entamoeba histolytica* ameba, and *A. lumbricoides* roundworm infection as risk factors for *N. americanus* hookworm co-infection reflects the high prevalence of parasitic infections in this population (*21*), in a setting with inadequate sanitary infrastructure despite improvement efforts (*36*). Why infections with these pathogens but not *S. stercoralis* roundworm (acquired similarly to hookworm) or *Giardia duodenalis* protozoa (acquired similarly to *E. histolytica* ameba) put refugees at risk for *N. americanus* infection deserves further study.

Female participants were less likely to acquire both *N. americanus* and *A. ceylanicum* hookworms, a finding that might reflect differential exposures, differences in immunity (*37*,*38*), differences in albendazole metabolism (*39*), or a combination of these factors. Although eosinophilia has been previously reported in experimental human infections with *A. ceylanicum* hookworm (*33*) and in *A. ceylanicum* case reports (*34*,*40*), a more striking eosinophilia was seen among those with *A. ceylanicum* infections than those with *N. americanus* infections. This observation supports the idea that, unlike the hookworms that only infect humans, the zoonotic *A. ceylanicum* parasite might be less able to downregulate the host’s IgE-mediated response to infection (*41*). No difference in hemoglobin levels was found between those with either hookworm species. Anemia is typically seen in *A. duodenale* infection and is less commonly associated with *N. americanus* infection (*3*); for *A. ceylanicum* infection, data on anemia are scant.

The treatment for *N. americanus* infection was only modestly effective (cure rate 42%–69%). It has been suggested that deworming might have enabled the zoonotic *A. ceylanicum* hookworm to fill a niche left by a decrease in anthropophilic hookworms (*32*). That *A. ceylanicum* infection was largely cured after single courses of treatment with albendazole (cure rate 92.8%–100%) is reassuring, although follow-up time periods in this evaluation were more varied than in most controlled studies specifically assessing response to treatment. Still, reinfection and newly acquired infection rates (0.58%–0.97% for *A. ceylanicum* and 2.3%–2.5% for *N. americanus*) were modest after deworming, compared with previous projections suggesting reinfection rates as high as 30% (*14*) after 3 months. With the average time between T1 and T2 exceeding 6 months (and the wide time range of 1–33 months), this population experienced a lower reinfection rate than has been suggested for endemic areas.

Persons who were infected with *N. americanus* hookworm at all 3 time points represent a group that might have never cleared the infection or might have cleared infection but were subsequently reinfected. However, the time between sample collections was not statistically different between those who were persistently positive and those who cleared infection after a single treatment (E.M. O’Connell, unpub. data). Reports of benzimidazole resistance are increasing in the literature on veterinary medicine; resistance in canine *Ancylostoma caninum* hookworm (*15*) and phylogenetically similar bovine intestinal nematodes (*17*) was associated with SNPs in the β-tubulin gene, particularly in codon 200 but also in codon 167 and, rarely, in codon 198. Likewise, other studies have shown low cure rates after benzimidazole administration in the setting of *N. americanus* infection, and research suggests that resistance is emerging (*42*). One group found that albendazole administration exerts selective pressure on *T. trichiura* codon 200 in Kenya and Haiti (*20*). The same group found that, in pooled *N. americanus* eggs from Haiti, the allele containing the resistant codon 200 had a mean allelic frequency of 36% (*43*). A frequency of 0% was found for the homozygous resistant genotype in hookworm eggs (species not identified) from Haiti and Panama (*20*), and although the authors reported a 2.3% frequency of the homozygous resistant genotype in hookworm eggs from Kenya, the frequency of the resistant genotype after treatment did not increase (*20*), raising questions about the significance of this allele. Our examination of codons 167 and 200 in samples persistently positive for *N. americanus* hookworm across all 3 time points, and of codon 200 in those with high levels of *N. americanus* DNA in fecal samples at any time point, revealed only homozygous wild-type β-tubulin genes.

Several possibilities might explain why β-tubulin mutations were not found to account for persistent *N. americanus* infections. First, the lesser-known codon 198 or another codon within the β-tubulin gene might be responsible for resistance in the hookworms infecting these refugees. Second, whereas amplification rates for our qPCR assay were as high as or higher than those in most other reports, up to 54% of samples positive for *N. americanus* DNA at T3 did not amplify adequately to determine genotype. Therefore, mutations in SNPs at either codon 167 or 200 could have been missed. Last, β-tubulin might not be the only gene involved in drug resistance and responsible for low cure rates in *N. americanus*. In this population, 88.5% (23 of 26) of those persistently positive for *N. americanus* at all 3 time points were male. The peak concentration in serum and area under the serum concentration time curve for albendazole sulfoxide and albendazole sulfone (the main active metabolites of albendazole) have been found to be higher in female than in male volunteers (*39*). Also, male refugees were possibly more likely than female refugees to quickly reacquire infection due to differences in environmental exposures.

One lesson from this evaluation was that unbiased surveys are necessary before pursuing highly specific molecular techniques when profiling organisms that infect a population, particularly when considering emerging infectious diseases. Although the use of the highly repetitive genomic sequence for *A. duodenale* hookworm is superior in sensitivity and specificity than the internal transcribed spacer 2 region (*44*), targeting the more conserved genomic region ultimately enabled detection of an unexpected organism, *A. ceylanicum* hookworm, which otherwise would have been missed in this population.

This project had several limitations. Control and nontreatment groups were absent. Participation was completely voluntary. The number of fecal sample collections for the third time point dropped off substantially because of logistical issues relating to collecting and shipping samples from the various US states after resettlement. Unlike other studies focused on *A. ceylanicum* hookworm, this evaluation did not include surveying the local cat and dog populations to establish potential reservoirs, which might be a worthwhile future research direction.

In summary, this cohort of US-bound refugees living in 3 camps in Thailand on the Myanmar–Thailand border was found to have a high prevalence of *N. americanus* hookworm with suboptimal cure rates after albendazole administration that do not seem to be attributable to mutations in the β-tubulin gene at codons 200 or 167. In addition, *A. ceylanicum* hookworm was the only other hookworm species identified in this population. *A. ceylanicum* infection had a much higher cure rate after a single course of albendazole, and those with *A. ceylanicum* monoinfection had a similar hemoglobin level as those with *N. americanus* monoinfection. Future mapping efforts of soil-transmitted helminths should take into account the emergence of *A. ceylanicum* hookworm infection in humans to further understand its distribution across the world. The recognition of the increased importance of zoonotic *A. ceylanicum* hookworm over that of *A. duodenale* hookworm in some populations raises epidemiologic questions about transmission dynamics and the differential effect on local health of these 2 species.

Technical Appendix Description of methods and restriction fragment length polymorphism of fecal samples from US-bound Myanmar refugees, Thailand, 2012–2015.
