# Supplementary material for: Ancylostoma ceylanicum Hookworm in Myanmar Refugees, Thailand, 2012–2015
Source: Emerg Infect Dis. 2018 Aug;24(8):1472–81. doi: 10.3201/eid2408.180280 (PMC6056130; doi:10.3201/eid2408.180280)
Supplement: Technical Appendix — Description of methods and restriction fragment length polymorphism of fecal samples from US-bound Myanmar refugees, Thailand, 2012–2015. [file 18-0280-Techapp-s1.pdf]

Article DOI: https://doi.org/10.3201/eid2408.180280

Ancylostoma ceylanicum Hookworm in Myanmar Refugees, Thailand, 2012–2015

Technical Appendix

Subject Recruitment and Sample Collection

A convenience sample of 2,004 US-bound refugees who lived in 3 camps (Mae La [camp 1], Mae Ra Ma Luang [camp 2], Mae La Oon [camp 3]) on the Myanmar–Thailand border provided written consent to participate in this evaluation (*1*). Fecal samples were collected at 3 time points (Figure 1 of main text): time point 1 (T1) was the time the refugee underwent the required overseas medical examination for resettlement into the United States (*2*), T2 was days to weeks before departure from the camp; and T3 was within 3 months after arrival in the United States. Per Centers for Disease Control and Prevention guidelines for presumptive treatment of parasites in US-bound refugees, all refugees were offered albendazole 200–400 mg (depending on age) for soil-transmitted helminths and ivermectin 200 mcg/kg daily for 2 days for strongyloidiasis. Presumptive treatment was given as directly observed therapy predeparture (doses at T1 and T2). Excluded from albendazole treatment were pregnant women and children <1 year of age, and excluded from ivermectin treatment were pregnant women and children weighing <15 kg (*3*).

DNA Extraction from Fecal Samples

Fecal samples were processed at the Centers for Disease Control and Prevention, Division of Parasitic Diseases and Malaria (Atlanta, Georgia, USA), and frozen at −80°C until arrival at the National Institutes of Health, where the samples remained frozen at −80°C until DNA extractions were performed. We performed DNA extraction from fecal samples as previously described (*4*). We spiked samples with an internal control plasmid (*5*) for downstream amplification using real-time quantitative PCR (qPCR) to verify successful extraction of DNA from fecal samples and exclude false-negative results.

Single-Nucleotide Polymorphism Detection in *Necator americanus*–Positive Samples

We tested additional samples from group members who were cured and had *N. americanus* DNA >150 pg/µL (on the basis of the standard curve) for single-nucleotide polymorphism (SNP) 200 using allelic discrimination primers and probes for SNP167 and SNP200.

We ran all samples plus positive and negative controls in 10-µL reactions with TaqMan Fast Advanced Master Mix 2x buffer (ThermoFisher Scientific, Waltham, MA, USA). By using the Viia 7 Real-Time PCR system (ThermoFisher Scientific), an initial 95°C 20-second incubation was followed by 2-step PCR cycling between 95°C for 1 second and 60°C for 20 seconds for 55 cycles. Each sample was run in triplicate. We constructed wild type (WT) and mutant plasmids as positive controls (Integrated DNA Technologies, Skokie, IL, USA) for each SNP and used a 50:50 mix of plasmids for heterozygote–positive controls. Positive controls were run in three 10-fold dilutions. On the basis of positive-control cycle threshold (C_T) results for all qPCR plates, rules were created to define SNP200 WT (mutant C_T – WT C_T >2), SNP200 mutant (WT C_T – mutant C_T >3), and SNP200 heterozygote (mutant C_T – WT C_T is between –1 and 1). Similar rules were created to define SNP167 WT (mutant C_T – WT C_T >7), SNP167 mutant (WT C_T – mutant C_T >4), and SNP167 heterozygotes (mutant C_T – WT C_T is between –1.5 and 1.5).

Statistical Analyses

Odds ratios of risk factors for infection with *Ancylostoma ceylanicum* and *N. americanus* were determined by using a generalized linear model that used over dispersion with binomial distribution and logit-link, and maximum likelihood was performed by using JMP 12.0.1 (https://www.jmp.com/en_us/home.html). These models tested the following parameters: sex; camp (1–3); age (infants and toddlers <2 years of age, children 2–18 years of age, adults >18 years of age); and co-infections with other soil-transmitted helminths (*Trichuris trichiura* roundworm, *Ascaris lumbricoides* roundworm, *Strongyloides stercoralis* roundworm, *N. americanus* hookworm, *A. ceylanicum* hookworm) and protozoa (*Cryptosporidium parvum*, *C. hominis*, *Entamoeba histolytica*, *Giardia duodenalis*).

References

1. Easton AV, Oliveira RG, O’Connell EM, Kepha S, Mwandawiro CS, Njenga SM, et al. Multi-parallel qPCR provides increased sensitivity and diagnostic breadth for gastrointestinal parasites of humans: field-based inferences on the impact of mass deworming. Parasit Vectors. 2016;9:38. PubMed http://dx.doi.org/10.1186/s13071-016-1314-y

2. Basuni M, Muhi J, Othman N, Verweij JJ, Ahmad M, Miswan N, et al. A pentaplex real-time polymerase chain reaction assay for detection of four species of soil-transmitted helminths. Am J Trop Med Hyg. 2011;84:338–43. PubMed http://dx.doi.org/10.4269/ajtmh.2011.10-0499

3. Pilotte N, Papaiakovou M, Grant JR, Bierwert LA, Llewellyn S, McCarthy JS, et al. Improved PCR-based detection of soil transmitted helminth infections using a next-generation sequencing approach to assay design. PLoS Negl Trop Dis. 2016;10:e0004578. PubMed http://dx.doi.org/10.1371/journal.pntd.0004578

4. George S, Kaliappan SP, Kattula D, Roy S, Geldhof P, Kang G, et al. Identification of *Ancylostoma ceylanicum* in children from a tribal community in Tamil Nadu, India using a semi-nested PCR-RFLP tool. Trans R Soc Trop Med Hyg. 2015;109:283–5. PubMed http://dx.doi.org/10.1093/trstmh/trv001

5. Lang AH, Drexel H, Geller-Rhomberg S, Stark N, Winder T, Geiger K, et al. Optimized allele-specific real-time PCR assays for the detection of common mutations in *KRAS* and *BRAF*. J Mol Diagn. 2011;13:23–8. PubMed http://dx.doi.org/10.1016/j.jmoldx.2010.11.007

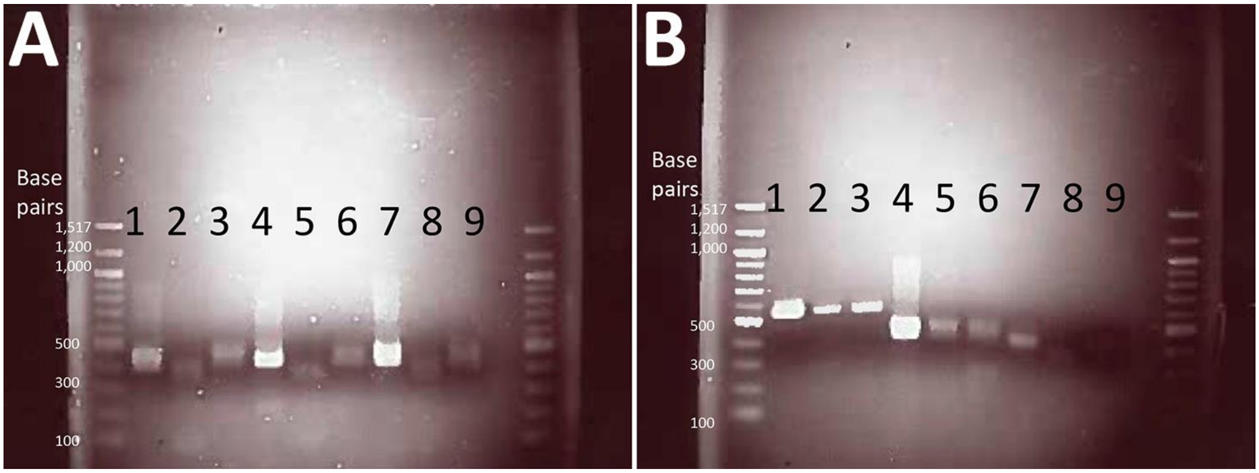

Technical Appendix Figure. Restriction fragment length polymorphism PCR analysis of fecal samples from Myanmar refugees, Thailand, 2012–2015. A) Identification of *Ancylostoma ceylanicum* hookworm from fecal samples from 2 refugees (refugee 1, lanes 1–3; refugee 2, lanes 4–6) positive by quantitative PCR with the Ad1 primer-probe set and negative with the Ad2 primer-probe set. Positive control was *A. ceylanicum* genomic DNA (lanes 7–9). Agarose gel (1.5%) of *Mva*I digestion (lanes 2, 5, 8), *Psp*1406I digestion (lanes 3, 6, 9), and uncut (lanes 1, 4, 7). *Mva*I digestion yielded bands 340 bp and 64 bp (lanes 2, 5, 8). Lack of digestion with *Psp*1406I is the pattern seen with *A. ceylanicum* and not *A. duodenale* DNA. B) Genomic *Necator americanus* (lanes 1–3) and *A. duodenale* DNA (lanes 4–6) run uncut (lanes 1, 4), subjected to *Mva*I digestion (lanes 2, 5), and subjected to *Psp*1406I digestion (lanes 3, 6). *N. americanus* DNA was not digested by either enzyme; bands are >500 bp. *A. duodenale* DNA was cut during the *Psp*1406I digestion, yielding a faint band at 250 bp (lane 6). Another unknown is pictured in lane 7.
